# Meningococcal disease epidemiology – United States, 2020–2023

**DOI:** 10.1371/journal.pone.0356184

**Published:** 2026-08-31

**Authors:** Amy B. Rubis, Basanta R. Wagle, Shreya Khuntia, Carolyn I. Wiest, Sandip Shrestha, Vishakh Walia, Rebecca L. Howie, Sara L. Eisen, Brittney Z. Thomas, Daya N. Marasini, Shalabh Sharma, Lucy A. McNamara

**Affiliations:** 1 Meningitis, Pertussis, and Diphtheria Epidemiology Branch, Division of Bacterial Diseases, National Center for Immunization and Respiratory Diseases, Centers for Disease Control and Prevention, Atlanta, Georgia, United States of America; 2 Great Hill Solutions, Atlanta, Georgia, United States of America; 3 Applied Science Research and Technology, Atlanta, Georgia, United States of America; 4 Meningitis, Pertussis, and Diphtheria Laboratory Branch, Division of Bacterial Diseases, National Center for Immunization and Respiratory Diseases, Centers for Disease Control and Prevention, Atlanta, Georgia, United States of America; University of Adelaide School of Medical Sciences: The University of Adelaide Adelaide Medical School, AUSTRALIA

## Abstract

**Background:**

High quality surveillance data are important to monitor changes in incidence and better understand risk factors for meningococcal disease. This report summarizes meningococcal disease epidemiology from 2020 through 2023 in the U.S.

**Methods:**

Epidemiologic information and isolates obtained through Enhanced Meningococcal Disease Surveillance for meningococcal disease cases reported through the National Notifiable Diseases Surveillance System (NNDSS) during 2020**–**2023 were included in the analysis. Isolates were characterized by serogrouping and molecular typing using whole genome sequencing.

**Results:**

A total of 1,192 confirmed and probable meningococcal disease cases were reported through NNDSS during 2020–2023. Incidence was highest in 2023 (0.13 cases per 100,000) and lowest in 2021 (0.06 cases per 100,000). The proportion of cases that were serogroup Y increased over time, accounting for 56.8% of cases in 2023 compared to 16.0% in 2021. For serogroup Y, the most common clonal complex (CC) was CC23 in 2020–2021 and CC174 in 2022–2023. Prior to 2022, CC174 was rare in the U.S., accounting for less than 10% of serogroup Y cases, but in 2022–2023, CC174 accounted for more than 60% of serogroup Y cases. Nine outbreaks began in 2020–2023 and included 109 cases. The proportion of cases associated with outbreaks ranged from 0.9% in 2020 to 26.2% in 2022.

**Conclusions:**

Meningococcal disease incidence continued to decline during the COVID-19 pandemic but increased to above pre-COVID-19 pandemic incidence by 2023. It will be important to continue to monitor meningococcal disease incidence, especially of serogroup Y, over the next several years.

## Background

In the United States, incidence of meningococcal disease, caused by the bacterium *Neisseria meningitidis*, had been declining and was at a historic low before the COVID-19 pandemic [1]. In 2019, overall meningococcal disease incidence was 0.11 cases per 100,000 population [2]. Serogroups B and C were the most common causes of disease, accounting for 26% and 23% of cases in 2019, with a combined incidence of 0.06 cases per 100,000 population. In addition, in 2019, penicillin- and ciprofloxacin-resistant serogroup Y meningococcal isolates were detected in the United States [3]; ongoing monitoring of this resistance will be important.

Despite the low incidence of meningococcal disease in the United States, outbreaks have been reported among college students, people experiencing homelessness, and men who have sex with men (MSM) [4–7]. Additional risk factors for meningococcal disease include age, certain underlying medical conditions (e.g., complement deficiency, asplenia, HIV infection), and certain medications (complement inhibitors) [8].

Since 2005, quadrivalent meningococcal conjugate (MenACWY) vaccines have been licensed and recommended by the Advisory Committee on Immunization Practices (ACIP) for routine use in persons aged 11–12 years, and a booster dose was recommended in 2010 for adolescents 16 years of age [9]. MenACWY vaccine is also recommended for persons at increased risk of meningococcal disease due to certain underlying medical conditions (e.g., complement deficiency, asplenia, HIV infection) and includes a primary series of two or more doses, depending on age, and booster doses every 3–5 years. Persons with increased exposure to *N. meningitidis* (e.g., microbiologists, populations at risk during outbreaks) are also recommended to receive MenACWY vaccine.

In 2015, ACIP recommended that individuals aged 16–23 years may be vaccinated with a serogroup B meningococcal (MenB) vaccine based on shared clinical decision-making [9]. ACIP also recommends that persons aged ≥10 years at increased risk for serogroup B disease because of certain underlying medical conditions (e.g., complement deficiency, asplenia) or increased exposure to *N. meningitidis* (e.g., microbiologists, populations at risk during outbreaks) receive MenB vaccine.

High quality surveillance data are important to monitor changes in incidence and better understand risk factors for meningococcal disease. During the COVID-19 pandemic, many countries saw declines in meningococcal disease and other infectious diseases, and in the post-pandemic period, a rebound in meningococcal disease cases has been reported [10–16]. This report summarizes meningococcal disease epidemiology from 2020 to 2023 in the United States during and following the COVID-19 pandemic.

## Methods

### Surveillance

Confirmed and probable meningococcal disease cases reported through National Notifiable Diseases Surveillance System (NNDSS) during 2020–2023 were included in the analysis. Cases were defined as confirmed or probable according to the Council of State and Territorial Epidemiologists’ surveillance case definition [17]. Confirmed cases were defined as isolation of *N. meningitidis* from a normally sterile body site (e.g., blood, cerebrospinal fluid [CSF]) or from purpuric lesions, or detection of *N. meningitidis*-specific nucleic acid in a specimen obtained from a normally sterile body site using a validated real-time polymerase chain reaction (rt-PCR) assay. A probable case was defined as detection of *N. meningitidis* antigen in formalin-fixed tissue by immunohistochemistry or in CSF by latex agglutination.

Meningococcal disease is nationally reportable in the United States through NNDSS. Limited epidemiologic data are collected through NNDSS, including basic demographic information (e.g., age, sex, race, and ethnicity), serogroup, and outcome. Additional epidemiologic data and isolates were collected through Enhanced Meningococcal Disease Surveillance (EMDS) for all 50 states and 3 large jurisdiction health departments. Additional epidemiologic data collected included serogroup and outcome to improve completeness; whether the case was part of a meningococcal disease outbreak; if the case was in a college student, man who has sex with men (MSM), person with HIV, or person experiencing homelessness (PEH); MenACWY and MenB vaccination history; and if the patient was taking a complement inhibitor (e.g., eculizumab or ravulizumab). All supplemental data collected through EMDS were linked to data submitted through NNDSS.

Meningococcal disease outbreaks were defined as 2–3 outbreak-associated cases within an organization during a 3-month period for organization-based outbreaks and multiple outbreak-associated cases with an incidence of meningococcal disease that is above the expected incidence in a community during a 3-month period for community-based outbreaks [18].

### Geographic Classification

County of residence of patients was classified into one of six urban-rural categories according to the 2013 National Center for Health Statistics Urban-Rural Classification Scheme [19]. To further classify counties into urban versus rural, four of these six categories (large central metropolitan, large fringe metropolitan, medium metropolitan, and small metropolitan) were combined into an urban category, and two (micropolitan and noncore) were combined into a rural category.

To examine geographic variation in serogroup distribution, cases were aggregated by HHS region. The 10 HHS regions group states as: Region 1 (Connecticut, Maine, Massachusetts, New Hampshire, Rhode Island, and Vermont), Region 2 (New Jersey, New York), Region 3 (Delaware, District of Columbia, Maryland, Pennsylvania, Virginia, and West Virginia), Region 4 (Alabama, Florida, Georgia, Kentucky, Mississippi, North Carolina, South Carolina, and Tennessee), Region 5 (Illinois, Indiana, Michigan, Minnesota, Ohio, and Wisconsin), Region 6 (Arkansas, Louisiana, New Mexico, Oklahoma, and Texas), Region 7 (Iowa, Kansas, Missouri, and Nebraska), Region 8 (Colorado, Montana, North Dakota, South Dakota, Utah, and Wyoming), Region 9 (Arizona, California, Hawaii, Nevada), and Region 10 (Alaska, Idaho, Oregon, and Washington). State-level analysis was not performed due to small case counts in many states.

### Laboratory methods

Serogrouping of *N. meningitidis* isolates was performed at state public health laboratories (SPHLs), then all available isolates from associated cases were submitted to CDC’s Bacterial Meningitis Laboratory as part of EMDS. At CDC, isolates were sequenced and further characterized by multilocus sequence typing [20] to determine sequence type (ST) and clonal complex (CC). Whole-genome sequencing (WGS) was conducted using libraries prepared with NEBNEXT Ultra II DNA library preparation kit (New England Biolabs, Ipswich, MA, USA) on an Illumina platform (Illumina Inc., San Diego, CA, USA). Pair-end Illumina sequencing reads were analyzed and characterized using the Bacterial Meningitis Genomic Analysis Platform (BMGAP) [21]. If serogroup result was unavailable from the SPHL or if WGS contradicted the SPHL’s serogroup result, slide agglutination was additionally performed to confirm the capsular polysaccharide expressed [22]. If an isolate was not available for submission to CDC, the SPHL’s serogroup result was used.

To investigate antimicrobial susceptibility, genomic assemblies from BMGAP were screened for the presence of antimicrobial resistance genetic determinants, including blaROB-1, blaTEM-1, PenA and PonA for penicillin, and *gyrA* and ParC for ciprofloxacin. Isolates carrying genetic determinants were then tested for confirmation of phenotypic presence of antimicrobial resistance by broth microdilution as described previously [23].

### Statistical analyses

Data were analyzed using SAS version 9.4 (SAS Institute Inc., Cary, North Carolina). Incidence rates were calculated using National Center for Health Statistics’ (NCHS) bridged-race postcensal population estimates for 2020 [24] and U.S. Census Bureau single-race population estimates for 2021–2023 [25]. Cases with unknown serogroup, race, or ethnicity were excluded from category-specific incidence calculation.

Proportions were calculated among reports with known responses. Differences in proportions were tested using Pearson’s χ2 or Fisher’s exact test. P values of <0.05 were considered statistically significant.

Case fatality ratios (CFRs) were calculated using cases with known outcome as the denominator. Because the epidemiology of meningococcal disease in the United States differed during and following the COVID-19 pandemic, data are presented by period for 2020–2021 as the years representing the COVID-19 pandemic and 2022–2023 as the years following the COVID-19 pandemic.

Choropleth maps of incidence and case counts by state were created using U.S. Census Bureau TIGER/Line Shapefiles (census.gov) in R version 4.5.0.

This activity was reviewed by CDC, deemed not research, and was conducted consistent with applicable federal law and CDC policy.^§^

## Results

**Fig 1:**
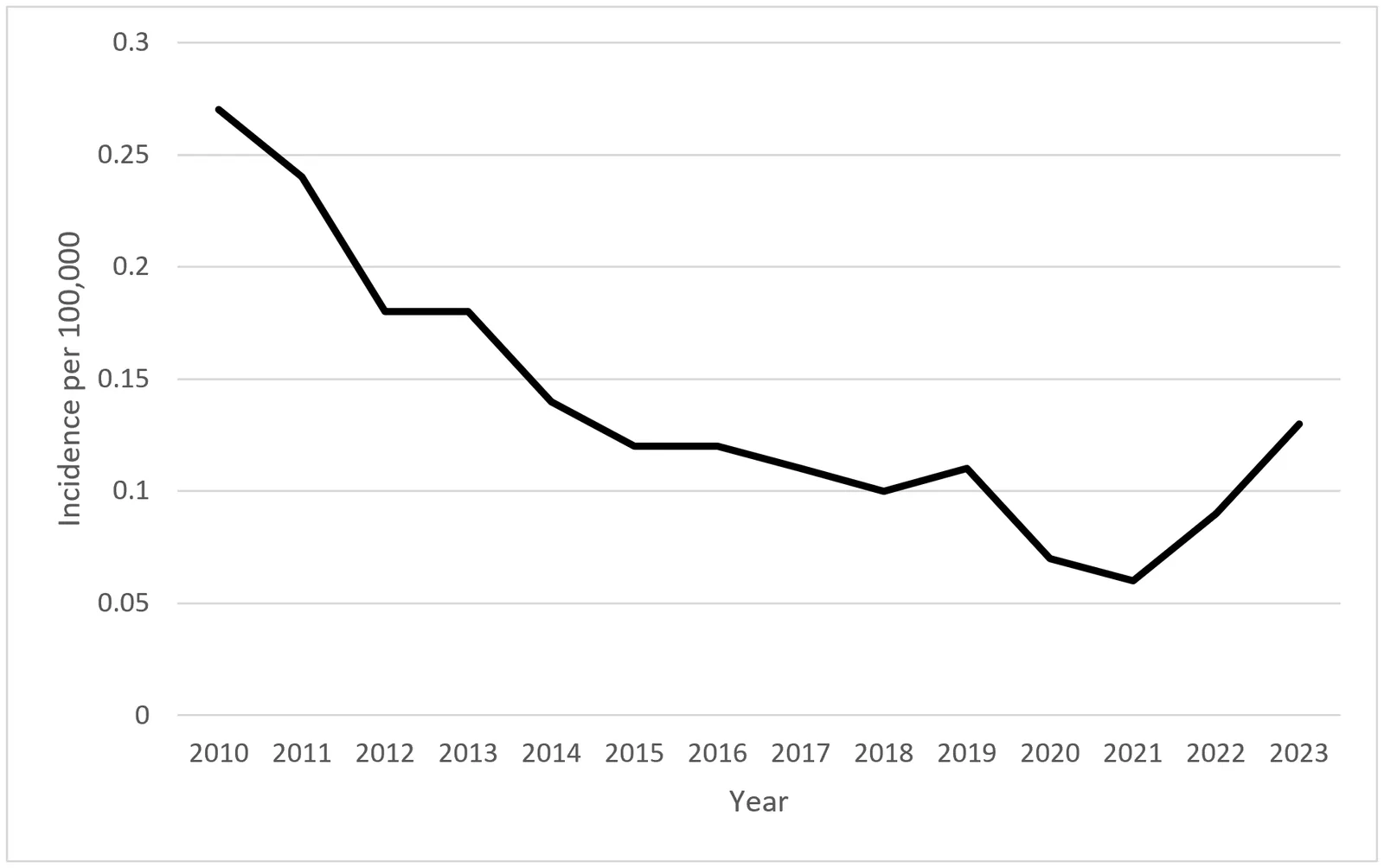
Incidence of Meningococcal Disease, United States, 2010–2023.

A total of 1,192 confirmed and probable meningococcal disease cases were reported through NNDSS from 2020–2023. Incidence was highest in 2023 (0.13 cases per 100,000) and lowest in 2021 (0.06 cases per 100,000) (Fig 1).

**Fig 2:**
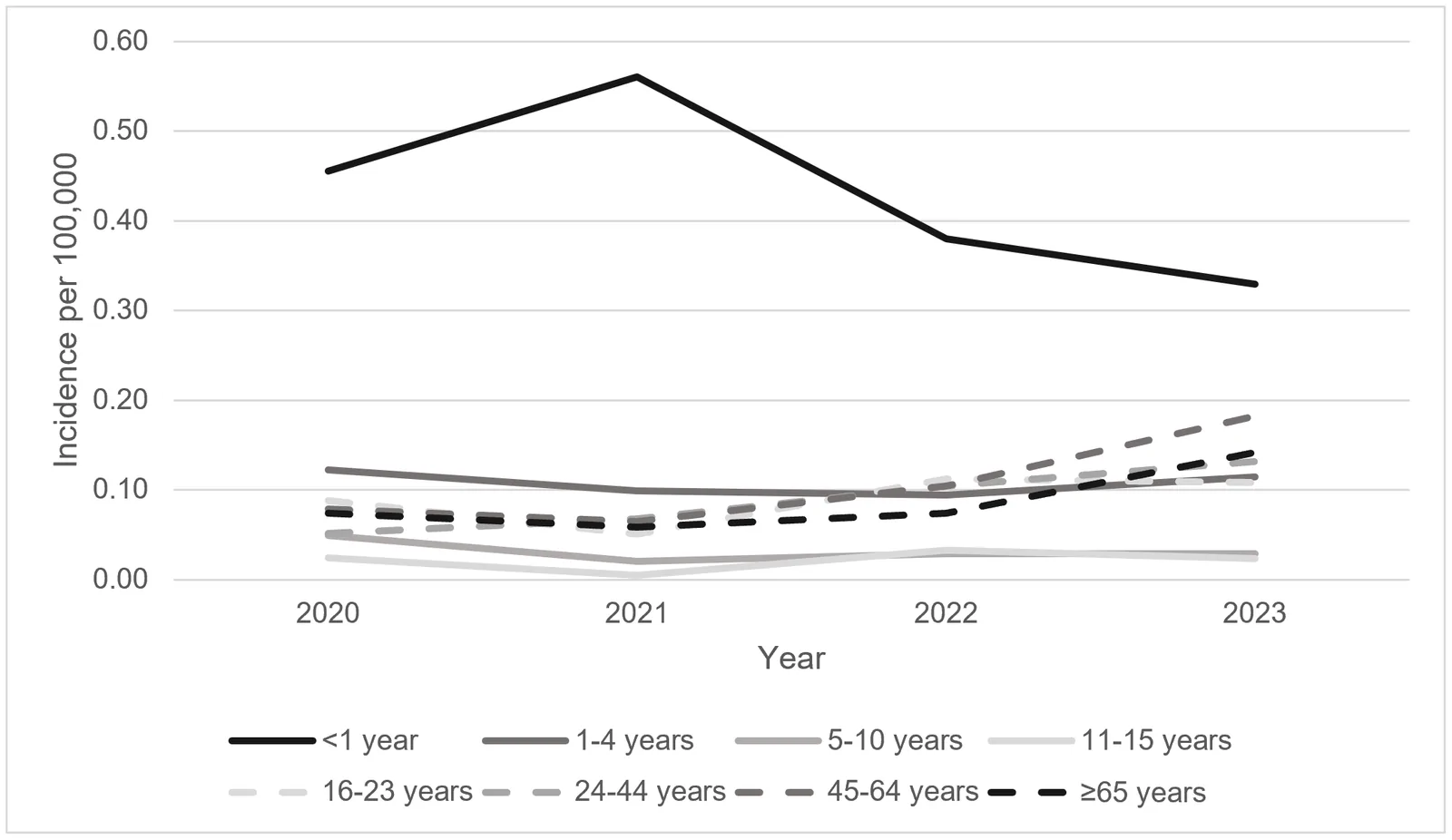
Incidence of Meningococcal Disease by Age Group, United States, 2020–2023.

**Fig 3:**
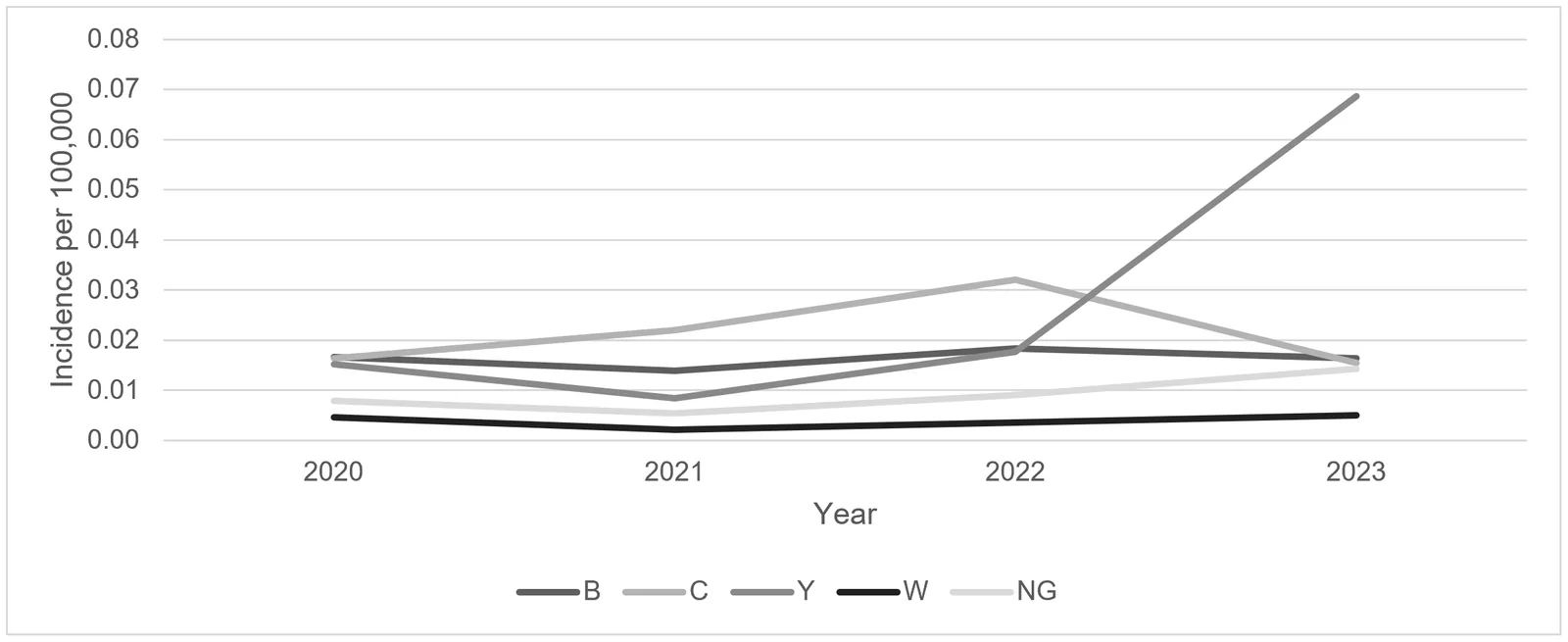
Incidence of Meningococcal Disease by Serogroup, United States, 2020–2023.

Incidence varied by age group and was highest among children aged <1 year in all years; however, incidence in this age group decreased over time (0.56 cases per 100,000 in 2021 and 0.33 cases per 100,000 in 2023) (Fig 2). Incidence in people aged ≥16 years increased in 2022 and 2023 compared to 2020 and 2021, with the largest increase among people aged 45–64 years (0.08 cases per 100,000 in 2020 to 0.18 cases per 100,000 in 2023). Trends in incidence also varied by serogroup; incidence of serogroups B, C, and W and nongroupable remained relatively consistent from 2020 to 2023, but serogroup Y incidence increased from 0.02 cases per 100,000 in 2020 to 0.07 cases per 100,000 in 2023 (Fig 3).

**Fig 4:**
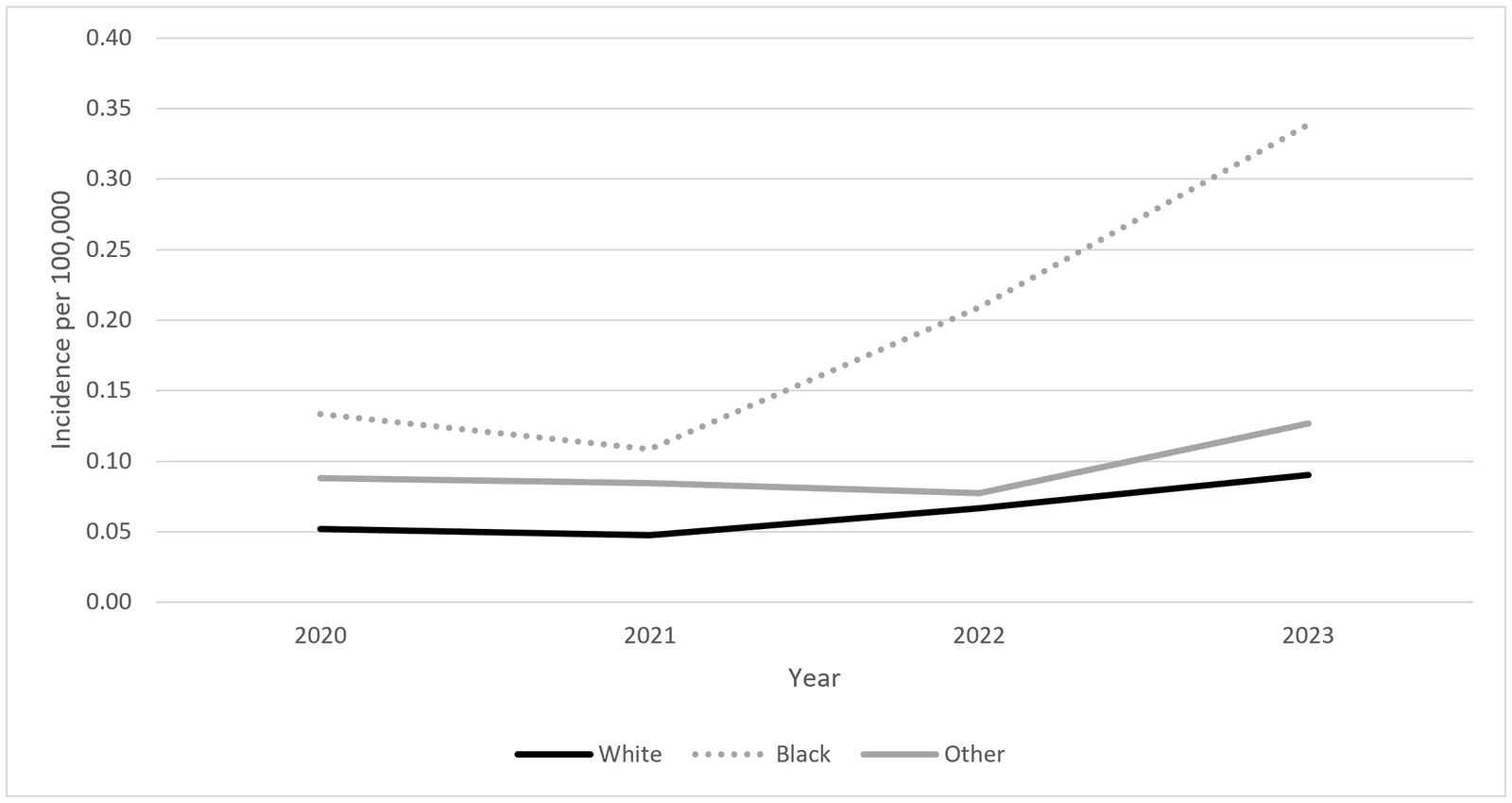
Incidence of Meningococcal Disease by Race―United States, 2020–2023.

Race was known for 1,138/1,192 (95.5%) patients. Of patients with known race, 645/1,138 (56.7%) cases occurred among White patients, 362 (31.8%) among Black or African American patients, and 131 (11.5%) among patients of other racial groups. Across years, incidence was consistently lowest among Asian/Pacific Islander persons, ranging from 0.02 cases per 100,000 in 2020 to 0.05 cases per 100,000 in 2023 (Fig 4). Incidence among all race groups increased from 2020 to 2023, with the largest increase observed among Black or African American persons (0.13 cases per 100,000 in 2020 to 0.34 cases per 100,000 in 2023).

**Fig 5:**
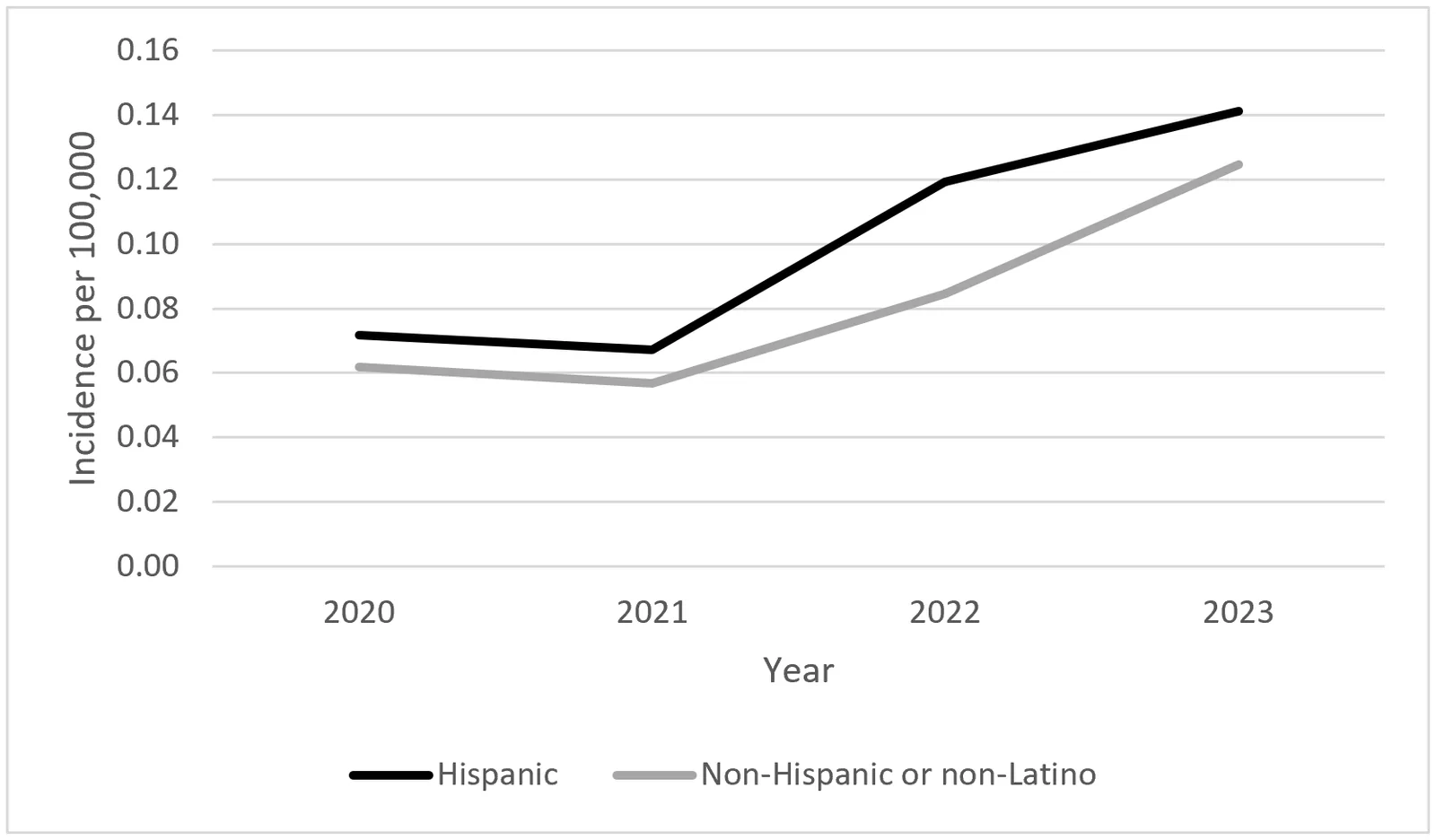
Incidence of Meningococcal Disease by Ethnicity―United States, 2020–2023.

Ethnicity was known for 1,137/1,192 (95.4%) patients. Of patients with known ethnicity, 254/1,137 (22.3%) cases occurred among Hispanic or Latino patients, and 883 (77.7%) cases occurred among non-Hispanic or non-Latino patients. Incidence among Hispanic or Latino persons was consistently higher than among non-Hispanic or non-Latino persons from 2020 to 2023 (0.07–0.14 cases per 100,000 and 0.06–0.13 cases per 100,000, respectively (Fig 5).

Several demographic, clinical, and risk factor characteristics of patients differed between 2022–2023 and 2020–2021 (Table 1 and Table 2). Compared with 2020–2021, a higher proportion of cases in 2022–2023 were outbreak associated (13.7% vs. 2.4%; p < .0001) among males (59.0% vs. 50.4%; p = 0.0046), had urban county of residence (90.9% vs. 86.4%; p = .0163), and in people with HIV (13.4% vs. 6.0%; p = .0034). In addition, the distribution of age (p = 0.0003), race (p = 0.0174), and clinical syndromes (p = 0.0039) differed by time period. Compared with 2020–2021, a higher proportion of patients in 2022–2023 were aged 24–44 years (29.8% vs. 24.9%), 45–64 years (31.6% vs. 26.7%), Black or African American (34.7% vs. 26.8%), and presented with bacteremia (46.7% vs. 38.9%).

**Table 1:** Characteristics of Meningococcal Disease Cases, United States, 2020–2021.

Characteristic	Serogroup B (n = 101)	Serogroup C (n = 127)	Serogroup W (n = 22)	Serogroup Y (n = 78)	Nongroupable (n = 44)	Other*/ Unknown Serogroup (n = 74)	Total
	N (%^†^)	N (%^†^)	N (%^†^)	N (%^†^)	N (%^†^)	N (%^†^)	N (%^†^)
Age Group (years)							
<1	22 (21.8)	3 (2.4)	0 (0)	5 (6.4)	2 (4.6)	5 (6.8)	37 (8.3)
1–4	9 (8.9)	11 (8.7)	0 (0)	4 (5.1)	2 (4.6)	8 (10.8)	34 (7.6)
5–10	4 (4.0)	3 (2.4)	1 (4.6)	5 (6.4)	1 (2.3)	3 (4.1)	17 (3.8)
11–15	2 (2.0)	0 (0)	0 (0)	1 (1.3)	1 (2.3)	2 (2.7)	6 (1.3)
16–23	13 (12.9)	6 (4.7)	2 (9.1)	3 (3.9)	12 (27.3)	12 (16.2)	48 (10.8)
24–44	20 (19.8)	39 (30.7)	4 (18.2)	17 (21.8)	16 (36.4)	15 (20.3)	111 (24.9)
45–64	22 (21.8)	44 (34.7)	8 (36.4)	23 (29.5)	5 (11.4)	17 (23.0)	119 (26.7)
≥65	9 (8.9)	21 (16.5)	7 (31.8)	20 (25.6)	5 (11.4)	12 (16.2)	74 (16.6)
Sex							
Male	48 (47.5)	68 (53.5)	8 (36.4)	37 (47.4)	18 (40.9)	46 (62.2)	225 (50.4)
Female	53 (52.5)	59 (46.5)	14 (63.6)	41 (52.6)	26 (59.1)	27 (36.5)	220 (49.3)
Race							
White	75 (78.1)	54 (44.6)	9 (47.4)	47 (63.5)	20 (48.8)	47 (70.2)	252 (60.3)
Black or African American	11 (11.5)	46 (38)	8 (42.1)	16 (21.6)	18 (43.9)	13 (19.4)	112 (26.8)
Other	10 (10.4)	21 (17.4)	2 (10.5)	11 (14.9)	3 (7.3)	7 (10.5)	54 (12.9)
Ethnicity							
Hispanic or Latino	11 (12.6)	26 (22.2)	1 (5.0)	36 (46.2)	5 (12.8)	7 (10.9)	86 (21.2)
Non-Hispanic or non-Latino	76 (87.4)	91 (77.8)	19 (95.0)	42 (53.9)	34 (87.2)	57 (89.1)	319 (78.8)
Clinical Syndrome							
Bacteremia	21 (23.3)	53 (47.8)	10 (52.6)	39 (52)	20 (48.8)	14 (20.6)	157 (38.9)
Meningitis	63 (70)	47 (42.3)	3 (15.8)	28 (37.3)	18 (43.9)	45 (66.2)	204 (50.5)
Bacteremic Pneumonia	2 (2.2)	9 (8.1)	3 (15.8)	4 (5.3)	1 (2.4)	2 (2.9)	21 (5.2)
Other	4 (4.4)	2 (1.8)	3 (15.8)	4 (5.3)	2 (4.9)	7 (10.3)	22 (5.4)
Died	11 (11.5)	19 (15.7)	6 (28.6)	7 (9.6)	5 (13.2)	5 (7.7)	53 (12.8)
County of Residence							
Rural	16 (16.3)	10 (8.2)	5 (22.7)	2 (2.6)	9 (20.5)	17 (23.9)	59 (13.6)
Urban	82 (83.7)	112 (91.8)	17 (77.3)	75 (97.4)	35 (79.6)	54 (76.1)	375 (86.4)
Outbreak Associated							
Yes	2 (2.0)	5 (4.2)	3 (13.6)	0 (0)	0 (0)	0 (0)	10 (2.4)
No	96 (98.0)	114 (95.8)	19 (86.4)	77 (100)	40 (100)	62 (100)	408 (97.6)
Experiencing homelessness							
Yes	4 (4.0)	16 (13.6)	1 (4.6)	1 (1.3)	1 (2.6)	3 (4.8)	26 (6.2)
No	95 (96.0)	102 (86.4)	21 (95.5)	76 (98.7)	38 (97.4)	60 (95.2)	392 (93.8)
College student (among 18–24-year-olds)							
Yes	6 (40.0)	0 (0)	0 (0)	1 (33.3)	2 (28.6)	6 (50.0)	15 (34.1)
No	9 (60.0)	5 (100)	2 (100)	2 (66.7)	5 (71.4)	6 (50.0)	29 (65.9)
MSM (among males ≥16 years old)							
Yes	0 (0)	7 (18.9)	1 (33.3)	4 (21.1)	2 (22.2)	1 (9.1)	15 (15.3)
No	19 (100)	30 (81.1)	2 (66.7)	15 (79)	7 (77.8)	10 (90.9)	83 (84.7)
Person with HIV							
Yes	2 (3.7)	7 (10.6)	0 (0)	5 (11.9)	0 (0)	0 (0)	14 (6.0)
No	52 (96.3)	59 (89.4)	12 (100)	37 (88.1)	27 (100)	31 (100)	218 (94.0)

Abbreviation: MSM, men who have sex with men

* Includes 1 serogroup A case and 1 serogroup E case

†Percent calculated of those with known response. Percent missing by variable: Race 18.4%, Ethnicity 9.2%, Clinical Syndrome 9.4%, Outcome (died) 7.2%, County of Residence 2.7%, Outbreak Associated 6.3%, Experiencing homelessness 6.3%, College student 10.2%, MSM 43.0%, HIV 48.0%

**Table 2:** Characteristics of Meningococcal Disease Cases, United States, 2022–2023.

Characteristic	Serogroup B (n = 116)	Serogroup C (n = 158)	Serogroup W (n = 29)	Serogroup Y (n = 289)	Nongroupable (n = 79)	Other*/ Unknown Serogroup (n = 75)	Total
	N (%^†^)	N (%^†^)	N (%^†^)	N (%^†^)	N (%^†^)	N (%^†^)	N (%^†^)
Age Group (years)							
<1	6 (5.2)	4 (2.5)	1 (3.5)	8 (2.8)	1 (1.3)	6 (8)	26 (3.5)
1–4	14 (12.1)	7 (4.4)	3 (10.3)	3 (1)	1 (1.3)	3 (4)	31 (4.2)
5–10	1 (0.9)	6 (3.8)	0 (0)	4 (1.4)	2 (2.5)	1 (1.3)	14 (1.9)
11–15	3 (2.6)	3 (1.9)	0 (0)	0 (0)	5 (6.3)	1 (1.3)	12 (1.6)
16–23	28 (24.1)	8 (5.1)	0 (0)	5 (1.7)	27 (34.2)	10 (13.3)	78 (10.5)
24–44	27 (23.3)	62 (39.2)	11 (37.9)	90 (31.1)	17 (21.5)	15 (20)	222 (29.8)
45–64	29 (25)	50 (31.7)	11 (37.9)	107 (37)	17 (21.5)	22 (29.3)	236 (31.6)
≥65	8 (6.9)	18 (11.4)	3 (10.3)	72 (24.9)	9 (11.4)	17 (22.7)	127 (17)
Sex							
Male	65 (56)	104 (65.8)	19 (65.5)	169 (58.5)	39 (49.4)	44 (58.7)	440 (59)
Female	51 (44)	54 (34.2)	10 (34.5)	120 (41.5)	40 (50.6)	31 (41.3)	306 (41)
Race							
White	89 (79.5)	87 (58.0)	10 (35.7)	119 (42.2)	46 (59.7)	43 (60.6)	394 (54.7)
Black or African American	10 (8.9)	48 (32.0)	13 (46.4)	136 (48.2)	21 (27.3)	22 (31)	250 (34.7)
Other	13 (11.6)	15 (10.0)	5 (17.9)	27 (9.6)	10 (13)	6 (8.5)	76 (10.6)
Ethnicity							
Hispanic or Latino	16 (14.2)	50 (32.3)	2 (6.9)	71 (24.7)	14 (18.2)	15 (21.1)	168 (23)
Non-Hispanic or non-Latino	97 (85.8)	105 (67.7)	27 (93.1)	216 (75.3)	63 (81.8)	56 (78.9)	564 (77)
Clinical Syndrome							
Bacteremia	45 (40.2)	70 (44.9)	13 (48.2)	157 (56.5)	36 (46.8)	16 (22.2)	337 (46.7)
Meningitis	63 (56.3)	71 (45.5)	9 (33.3)	68 (24.5)	33 (42.9)	42 (58.3)	286 (39.6)
Bacteremic Pneumonia	2 (1.8)	3 (1.9)	1 (3.7)	27 (9.7)	3 (3.9)	4 (5.6)	40 (5.5)
Other	2 (1.8)	12 (7.7)	4 (14.8)	26 (9.4)	5 (6.5)	10 (13.9)	59 (8.2)
Died	13 (11.4)	24 (15.5)	4 (14.3)	32 (11.1)	9 (11.7)	4 (5.6)	86 (11.7)
County of Residence							
Rural	21 (18.3)	6 (3.8)	3 (10.3)	19 (6.7)	5 (6.4)	13 (17.3)	67 (9.1)
Urban	94 (81.7)	152 (96.2)	26 (89.7)	263 (93.3)	73 (93.6)	62 (82.7)	670 (90.9)
Outbreak Associated							
Yes	8 (7)	44 (29.7)	14 (48.3)	30 (10.4)	1 (1.3)	3 (4.2)	100 (13.7)
No	106 (93)	104 (70.3)	15 (51.7)	258 (89.6)	78 (98.7)	69 (95.8)	630 (86.3)
Experiencing homelessness							
Yes	1 (0.9)	10 (6.5)	0 (0)	18 (6.4)	1 (1.3)	5 (7.5)	35 (4.8)
No	110 (99.1)	145 (93.6)	29 (100)	265 (93.6)	78 (98.7)	62 (92.5)	689 (95.2)
College student (among 18–24-year-olds)							
Yes	14 (56)	2 (20)	0 (0)	0 (0)	17 (63)	4 (44.4)	37 (49.3)
No	11 (44)	8 (80)	0 (0)	4 (100)	10 (37)	5 (55.6)	38 (50.7)
MSM (among males ≥16 years old)							
Yes	2 (5.7)	37 (49.3)	1 (9.1)	21 (18.6)	3 (12.5)	3 (17.7)	67 (24.4)
No	33 (94.3)	38 (50.7)	10 (90.9)	92 (81.4)	21 (87.5)	14 (82.4)	208 (75.6)
Person with HIV							
Yes	2 (3.9)	20 (16.5)	0 (0)	35 (18.6)	3 (6.0)	3 (7.9)	63 (13.4)
No	50 (96.2)	101 (83.5)	22 (100)	153 (81.4)	47 (94.0)	35 (92.1)	408 (86.6)

Abbreviation: MSM, men who have sex with men

* Includes 3 serogroup A cases and 1 serogroup E case

†Percent calculated of those with known response. Percent missing by variable: Race 3.5%, Ethnicity 1.9%, Clinical Syndrome 3.2%, Outcome (died) 1.6%, County of Residence 1.2%, Outbreak Associated 2.2%, Experiencing homelessness 3.0%, College student 0%, MSM 29.3%, HIV 36.9%

**Fig 6:**
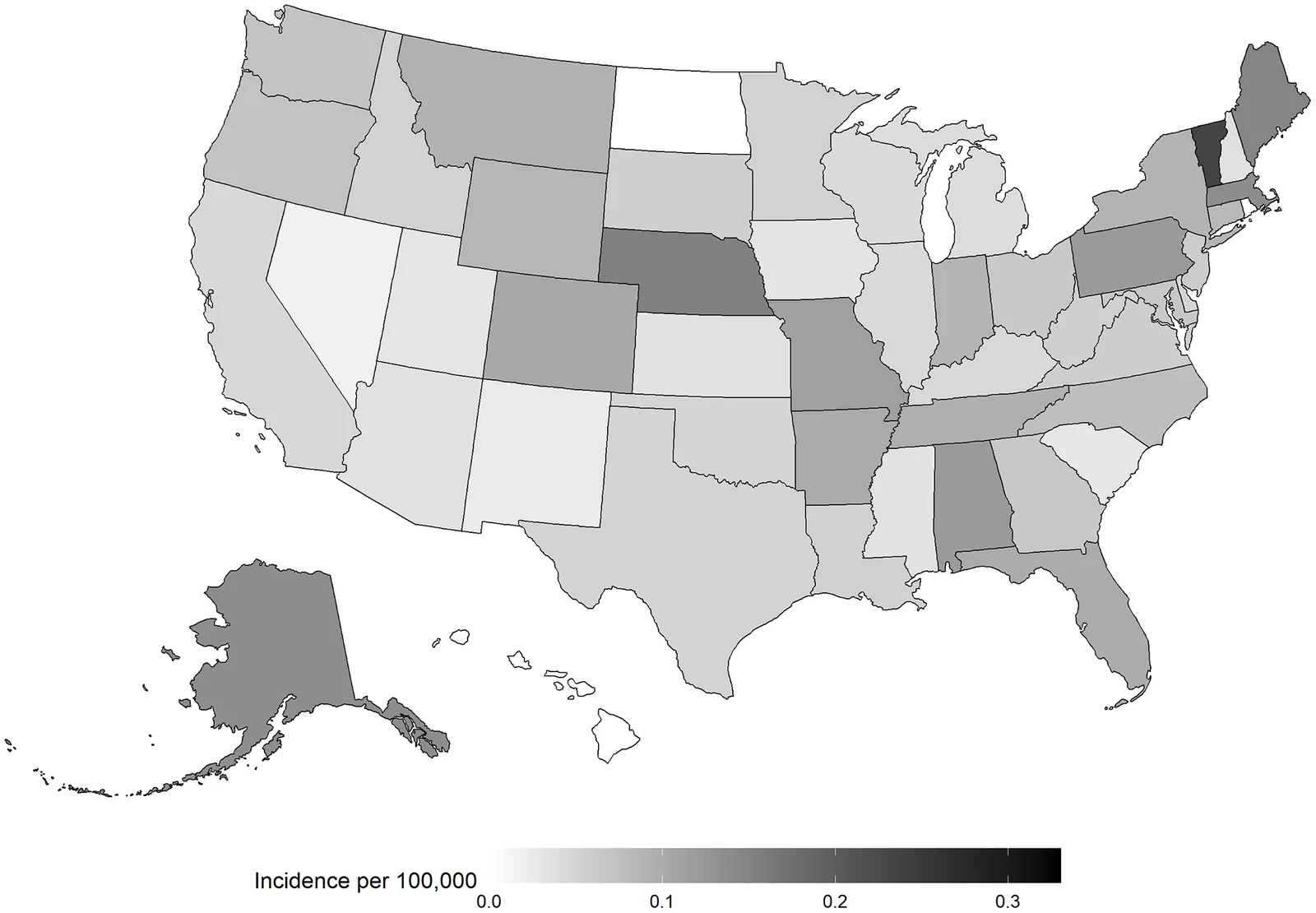
Incidence of Meningococcal Disease by State, United States, 2020–2021. Map created in R using public-domain TIGER/Line Shapefiles from the U.S. Census Bureau.

**Fig 7:**
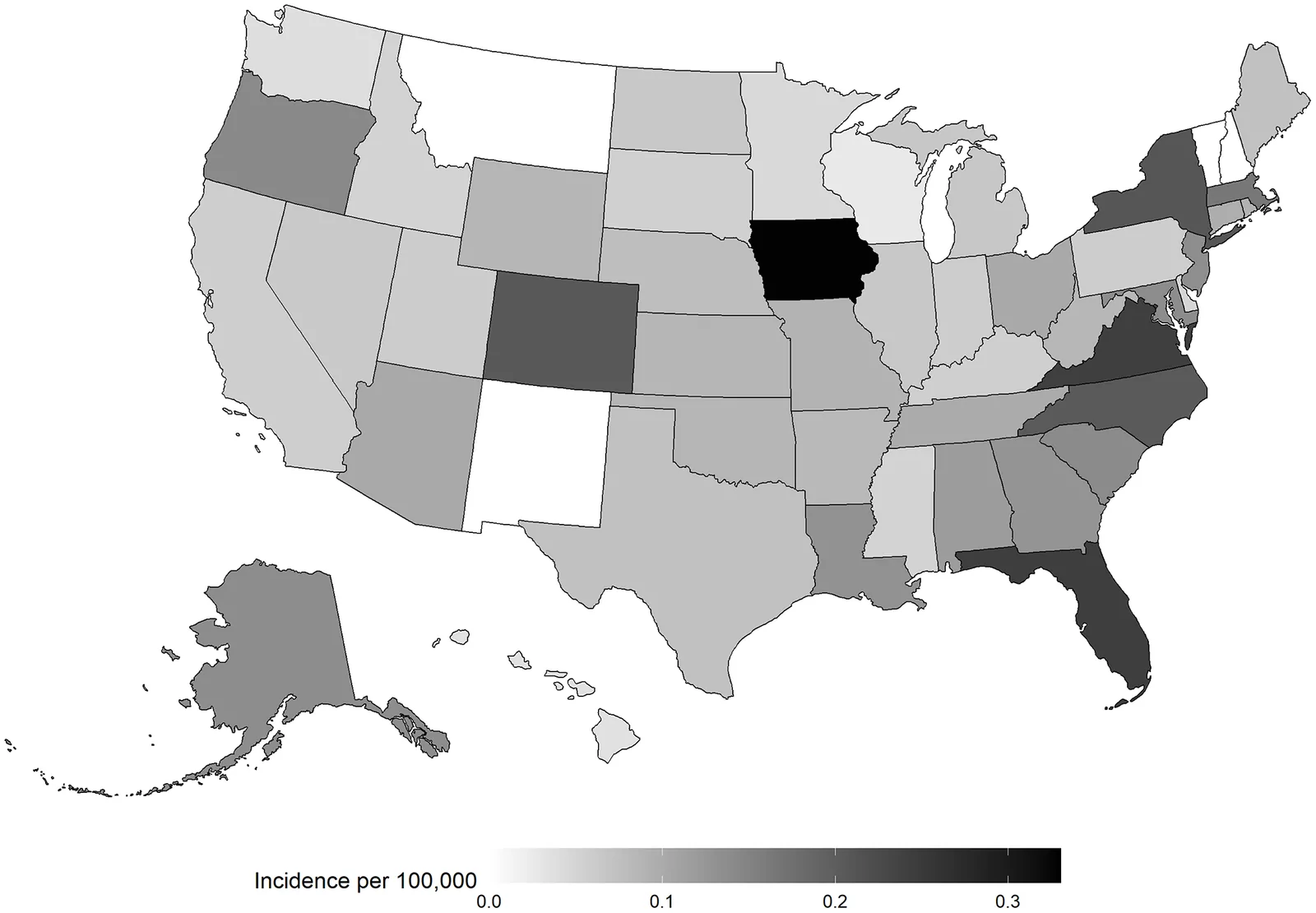
Incidence of Meningococcal Disease by State, United States, 2022–2023. Map created in R using public-domain TIGER/Line Shapefiles from the U.S. Census Bureau.

By state, incidence ranged from 0.00 to 0.24 cases per 100,000 in 2020–2021 and 0.00 to 0.33 cases per 100,000 in 2022–2023. (Fig 6 and Fig 7). In 2020–2021, the lowest incidence was observed in Washington, D.C., Hawaii, North Dakota, and Rhode Island (0.00 per 100,000 population); the highest incidence was observed Vermont (0.24 per 100,000 population). In 2022–2023, the lowest incidence was observed in Montana, New Hampshire, New Mexico, and Vermont (0.00 per 100,000 population); the highest incidence was observed Iowa (0.33 per 100,000 population).

### Vaccination history

MenACWY vaccination history was complete for 111/137 (81.0%) cases in people aged 16–24 years. Completeness varied by year with the lowest completeness in 2020 (68.8%) and the highest in 2023 (90.2%) The proportion of patients aged 16–24 years that received one or more doses of MenACWY vaccine decreased from 77.3% in 2020 to 66.7% in 2021, but increased in 2022 and 2023, with 81.1% of patients in 2023 having received MenACWY vaccine. Of the patients that received MenACWY vaccine, 8/83 (9.6%) had disease caused by serogroups A, C, W, or Y. Four of these 8 patients had only received a single dose of MenACWY vaccine. The remaining four patients received two doses of MenACWY vaccine, with the most recent dose more than 5 years before onset.

MenB vaccine history was complete for 91/137 (66.4%) patients aged 16–24 years. The proportion of patients who received one or more doses of MenB vaccine increased slightly over time from 20.0% in 2020 to 24.0% in 2023. Of the patients who received MenB vaccine, 4/22 (18%) had disease caused by serogroup B. One patient had received one dose of MenB-4C 6 months before onset, one received two doses of MenB-FHbp four years before onset, one received two doses of MenB-4C three years before onset, and one was a patient taking eculizumab who had received three doses of MenB-4C, with the most recent dose less than one month before onset.

### Laboratory results

Among 1,057/1,192 cases with known serogroup, serogroups B (13.6–26.8% of cases), C (12.8–41.7% of cases), and Y (16.0–56.8%) were predominant in all four years, with serogroup B most common in 2020, serogroup C most common in 2021 and 2022, and serogroup Y most common in 2023, when it accounted for 56.8% of cases.

**Fig 8:**
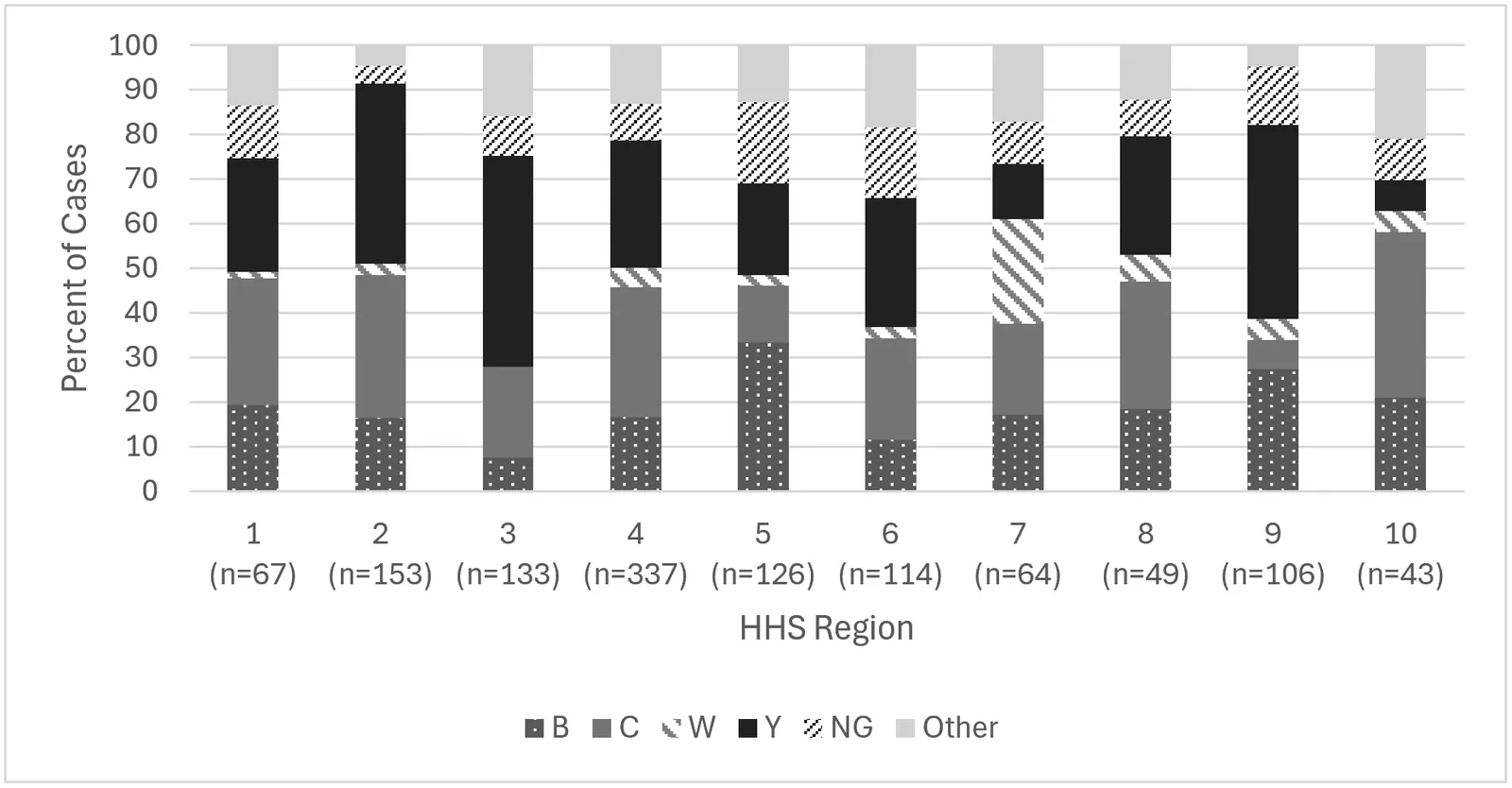
Serogroup Distribution by HHS region, United States, 2020–2023. The 10 HHS regions group states as: Region 1 (Connecticut, Maine, Massachusetts, New Hampshire, Rhode Island, and Vermont), Region 2 (New Jersey, New York), Region 3 (Delaware, District of Columbia, Maryland, Pennsylvania, Virginia, and West Virginia), Region 4 (Alabama, Florida, Georgia, Kentucky, Mississippi, North Carolina, South Carolina, and Tennessee), Region 5 (Illinois, Indiana, Michigan, Minnesota, Ohio, and Wisconsin), Region 6 (Arkansas, Louisiana, New Mexico, Oklahoma, and Texas), Region 7 (Iowa, Kansas, Missouri, and Nebraska), Region 8 (Colorado, Montana, North Dakota, South Dakota, Utah, and Wyoming), Region 9 (Arizona, California, Hawaii, Nevada), and Region 10 (Alaska, Idaho, Oregon, and Washington) Cases with unknown serogroup (n = 135) are excluded.

Serogroup distribution differed by HHS region (p < .0001) (Fig 8). The highest proportions of serogroup Y cases were seen in Region 3 (47.4%), Region 9 (43.4%), and Region 2 (40.5%); the lowest proportion of serogroup Y cases was seen in Region 10 (7.0%). Region 7 had the highest proportion of serogroup W cases (23.4%) compared to 0–6.1% in other regions. Region 10 had the highest proportion of serogroup C cases (37.2%). The highest proportion of serogroup B cases was seen in Region 5 (33.3%).

CC results were available for 827 isolates sequenced at CDC. For serogroup C, CC103 was the most common CC in 2020–2021 and 2023, but in 2022, CC11 was the most common, driven by a large serogroup C CC11 MSM outbreak described below (Supplemental Table 1). For serogroup Y, CC23 was the most common CC in 2020–2021, and CC174 was the most common CC in 2022–2023. Prior to 2022, CC174 was rare in the U.S. and accounted for less than 10% of serogroup Y cases, but in 2022 and 2023, CC174 accounted for more than 60% of serogroup Y cases.

**Fig 9:**
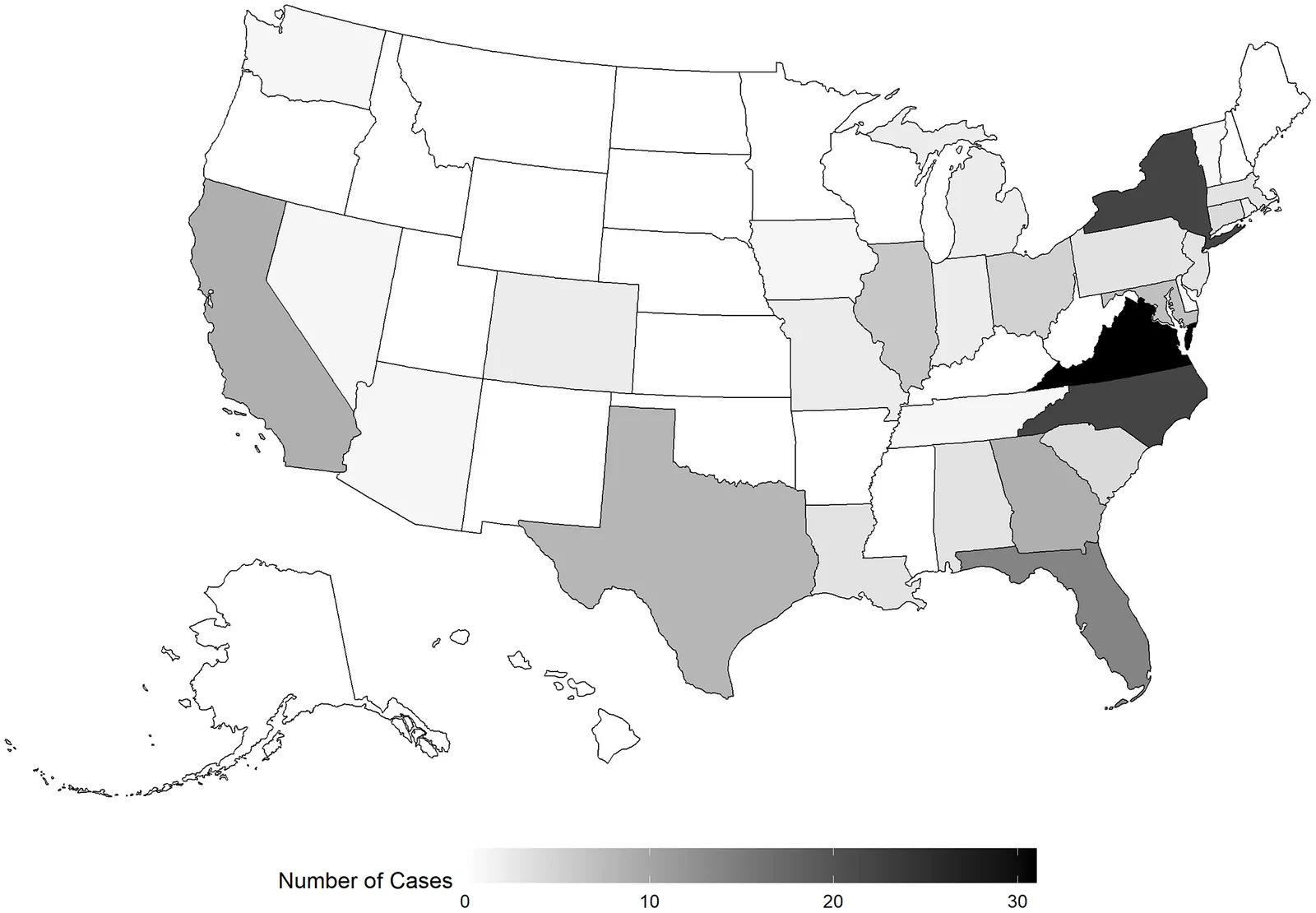
Serogroup Y ST-1466 Meningococcal Disease Cases by State, United States, 2020–2023. Map created in R using public-domain TIGER/Line Shapefiles from the U.S. Census Bureau.

Of the 185 serogroup Y CC174 cases, 174 (94.1%) were ST-1466, and many of these cases nationwide were genomically closely related. Of cases with known information, 16.8% (29/173) of serogroup Y CC174 ST-1466 cases were associated with outbreaks compared to 0% of other serogroup Y cases with known ST (0/156). All outbreak-associated ST-1466 cases were associated with a single community outbreak in Virginia, described previously [26]. ST-1466 cases were more concentrated on the East Coast but were identified across the country (Fig 9). The demographics of serogroup Y ST-1466 cases differed from other serogroup Y cases, with a larger proportion of serogroup Y ST-1466 cases among people aged 30–60 years (107/174, 61.5%) compared to other serogroup Y cases with known ST (69/156, 44.2%) (p < .0001). In addition, a larger proportion of ST-1466 cases were among Black or African American patients (106/171, 62.0%) compared to other serogroup Y cases with known ST (35/128, 27.3%) (p < .0001). A larger proportion of ST-1466 cases were among people with HIV (33/118, 28.0%) compared to other serogroup Y cases with known ST (6/93, 6.5%) (p < .0001). Of patients with serogroup Y CC174 ST-1466 cases with known outcome, 24/173 (13.9%) died; this CFR for other serogroup Y cases with known ST during this period was 7.3%. This difference was not statistically significant (p = .06). When stratified by age group, the CFR for serogroup Y CC174 ST-1466 cases remained higher than other serogroup Y cases (24–44-year-olds: 23.0% vs. 13.2%; 45–64-year-olds: 6.2% vs. 4.4%; ≥ 65-year-olds: 13.9% vs 7.3%) but the difference was not statistically significant (p = .23, p = .31, p = .06 respectively).

When the ST-1466 serogroup Y cases from the Virginia outbreak were excluded from analysis, the proportion of patients who were Black or African American remained higher in 2022–2023 compared 2020–2021 (32.6% vs. 26.8%) but the difference in the distribution of race between the two time periods was no longer statistically significant (p = 0.11). The differences in rural/urban county of residence (p = 0.0104), HIV (p = 0.0043), age distribution (p = 0.0011), and clinical syndrome (p = 0.0185) remained significant after the Virginia outbreak cases were excluded.

While the proportion of serogroup Y cases that were CC23 decreased over time, the overall number of serogroup Y CC23 cases increased from 32 in 2020 to 62 in 2023. This was driven by ST-3587 cases, the majority of which were resistant to penicillin (pen^R^) or to ciprofloxacin and penicillin (cipro^R^pen^R^). Of the 827 isolates sequenced at CDC, isolates from 49 cases in 2020–2023 were pen^R^ only and isolates from 45 cases were cipro^R^pen^R^. The number of pen^R^ ST-3587 isolates increased from 12/20 ST-3587 isolates (60.0%) in 2020 to 21/47 (44.7%) in 2023, and the number of cipro^R^pen^R^ ST-3587 isolates identified tripled from 8/20 (40.0%) in 2020 to 24/47 (46.8%) in 2023.

### Meningococcal disease outbreaks

**Fig 10:**
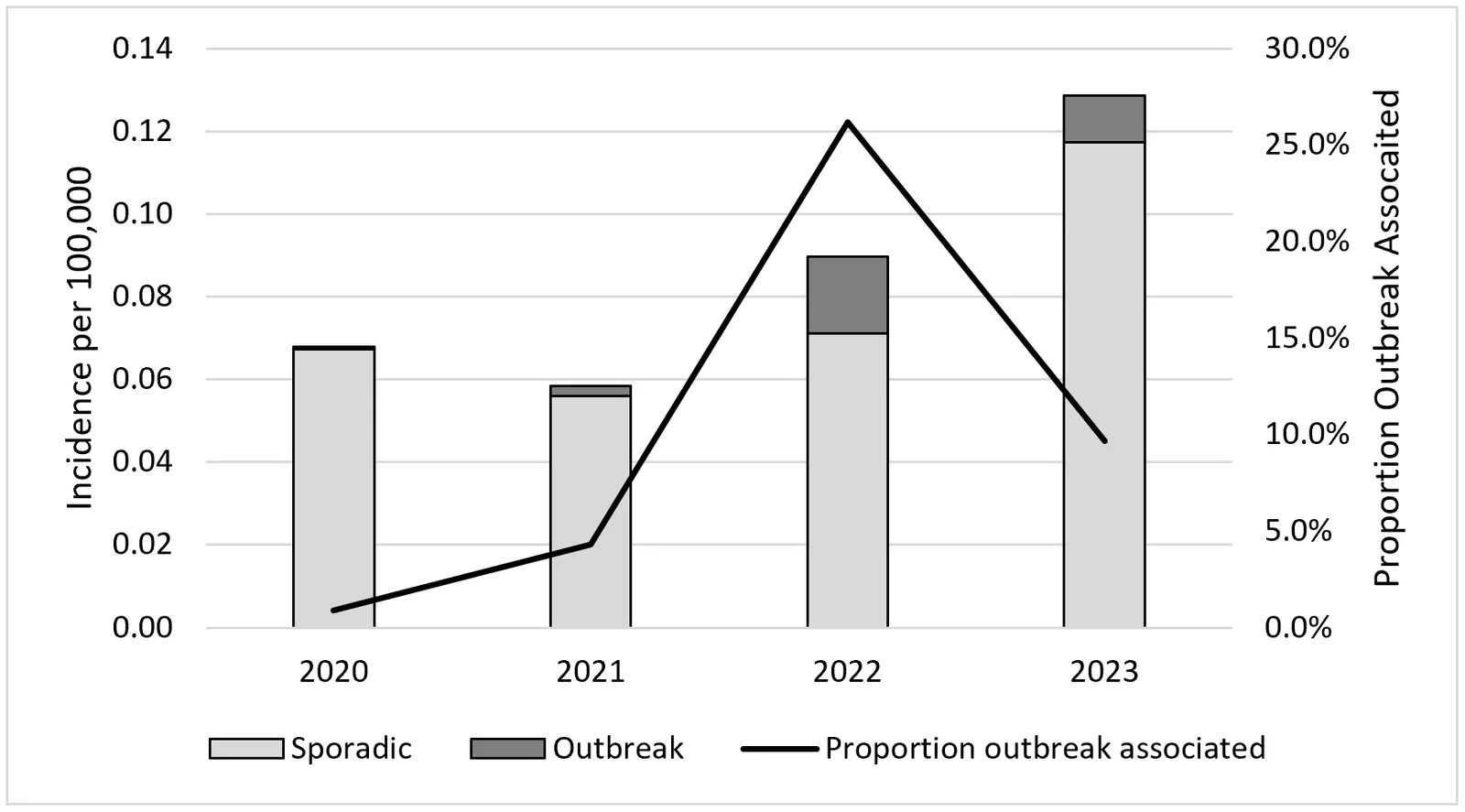
Incidence of meningococcal disease and proportion of cases that were outbreak associated, United States, 2020–2023.

Among 1,148 cases with known information, 110 (9.6%) cases were associated with an outbreak. Of these, 109 were part of 9 outbreaks that began in 2020–2023 (Supplemental Table 2). Two outbreaks had cases occur after the period being analyzed, and those additional cases are included in the total number of outbreak cases and deaths, the median number of cases, and the median duration. One additional outbreak-associated case reported in 2020 was related to an outbreak that began in 2019 among PEH. The proportion of cases associated with outbreaks ranged from 0.9% in 2020 to 26.2% in 2022 (Fig 10). Seven of the 9 outbreaks occurred in community settings, and two occurred in organizations. The community outbreaks included three outbreaks among PEH, one MSM outbreak, one statewide outbreak, and two other small community outbreaks. The organization-based outbreaks included a university outbreak and an outbreak in a county home.

The 7 community outbreaks included two serogroup B outbreaks, three serogroup C outbreaks, a serogroup W outbreak, and a serogroup Y outbreak. The organization-based outbreaks were serogroup W and serogroup B. The median number of cases in the community outbreaks was 6 compared to 2.5 for organization-based outbreaks. The median duration, or time between onset of the first and last case, for community outbreaks was 37 days (range 2–837 days), with three outbreaks being less than two weeks in duration and three outbreaks being over a year in duration. The two organization-based outbreaks were 38 and 52 days in duration.

There were several notable outbreaks during this period. The outbreak with the highest number of cases was a serogroup C CC11 outbreak among MSM in Florida, with a total of 45 cases [27]. The longest outbreak was a statewide serogroup Y ST-1466 CC174 outbreak in Virginia that was still ongoing at the end of 2023 and was declared over in February 2025 [26,28].

Three outbreaks were reported among PEH during this period. Two were serogroup C and one was serogroup B. The two serogroup C outbreaks were caused by different sequence types/clonal complexes. One outbreak was serogroup C CC103, and the second outbreak was serogroup C CC11. All three outbreaks had 2–3 cases and had a duration of less than 7 days.

## Discussion

Meningococcal disease incidence continued to decline during the COVID-19 pandemic but began to increase in 2022 and increased to above pre-COVID-19 pandemic incidence in 2023 (0.13 cases per 100,000), with the highest incidence observed since 2014 (0.14 cases per 100,000). While incidence remained highest in children aged <1 year, similar to 2015–2019, the difference in incidence between this age group and other age groups decreased substantially over time. While incidence was highest among Black or African American persons, similar to previous years, the difference in incidence between Black or African American persons and persons of other races increased in 2022 and 2023. Consistent with previous years, serogroups B, C, and Y were the most common causes of disease. The incidence of serogroup B decreased over time compared to 2015–2019, and the incidence of serogroup Y increased over time [29,30]. The incidence of serogroups C, W, and nongroupable cases remained relatively consistent over time. The proportion of meningococcal disease cases that were previously vaccinated with either MenACWY vaccine or MenB vaccine decreased during the COVID-19 pandemic period compared to 2019 [30] but has since increased in the post-COVID-19 pandemic period (2022–2023).

During the post-COVID-19 pandemic period, there were several notable differences compared to pre-COVID-19 pandemic meningococcal disease epidemiology from 2015 through 2019 [30]. More cases were associated with an outbreak post-COVID-19 pandemic compared to pre-pandemic (13.7% vs. 8.7%), which was driven by 2 large outbreaks in the post-pandemic period [30]. The outbreaks that occurred from 2020–2023 also differed from outbreaks in previous years [31], Cooper et al. [Unpublished]]; only one university outbreak occurred compared to 12 university outbreaks in the preceding 6 years. The absence of university outbreaks specifically in 2020−21 is likely related to COVID-19 pandemic-related restrictions on in person university classes and activities, but it is unclear if this trend will continue. The age distribution also differed, with more cases in people aged 24−64 and fewer infant cases post-COVID-19 pandemic. A larger proportion of cases were among Black or African American persons (34.7% vs. 17.4%) and Hispanic persons (23.0% vs. 17.0%) in the post-pandemic period compared to pre-COVID-19 pandemic. The proportions of cases among PEH, college students, and MSM were similar pre- and post-COVID-19 pandemic.

The increase in incidence observed during 2020–2023 was primarily driven by serogroup Y, particularly serogroup Y CC174/ST-1466, which previously had been rare in the United States, accounting for 1–6 serogroup Y cases each year from 2015–2019 [30]. The increase in this specific strain thus seems unlikely to be simply a rebound following the COVID-19 pandemic, but rather an expansion of a new strain in the population. Expansions of new meningococcal strains in a population are not unprecedented and have been seen previously in the United Kingdom and the Netherlands with serogroup W CC11 and in Nigeria and Burkina Faso with serogroup C CC10217 [32–36]. Serogroup Y incidence in 2023 reached the highest incidence observed since 2011 (0.13 cases per 100,000) [37]. The increase in serogroup Y ST-1466 cases correlates with disparities in incidence among people with HIV and Black or African American persons compared to other racial groups. While the majority of the serogroup Y cases were CC174/ST-1466, there was also an increase in CC23/ST-3587 pen^R^ and cipro^R^pen^R^ isolates from cases observed during 2020–2023, which led to updated guidance for prophylaxis around meningococcal disease cases in areas with ciprofloxacin resistance [38].

EMDS is a crucial part of national meningococcal disease surveillance and provides information on key variables for understanding meningococcal disease epidemiology, but limitations in the data exist. Completeness of some variables did decrease during the COVID-19 pandemic compared to pre-pandemic years, including serogroup, outcome, MSM, and college attendance. In addition, there are still consistent gaps in completeness for some variables, specifically whether the case was in a MSM or a person with HIV, so it is possible that some patients are being misclassified, e.g., as non-MSM due to missing data. These gaps in completeness for these variables are likely related to the sensitive nature of this data collection. Also, while information was available on how many outbreaks occurred and the type of outbreak, complete information on affected populations and outbreak response details presented in previous analyses [31], Cooper et al. [Unpublished]] were not available for all outbreaks during this period.

Meningococcal disease incidence declined sharply during the COVID-19 pandemic, similar to what was seen in other countries [11], decreasing to an all-time low incidence of 0.06 cases per 100,000 in 2021. In 2022, incidence began to return to pre-pandemic levels and continued to increase in 2023 to the highest incidence observed since 2014. This increase in incidence was driven largely by the introduction of a new serogroup Y ST-1466 strain, resulting in a different incidence distribution compared to previous years, and increased incidence among Black or African American persons compared to other racial groups. It is unclear how the trajectory of meningococcal disease incidence will change moving forward with some of this uncertainty being driven by the emergence of the ST-1466 strain. As such, it will be important to continue to monitor incidence and better understand risk factors for meningococcal disease.

^§^See, e.g., 45 C.F.R. part 46.102(l)(2), 21 C.F.R. part 56; 42 U.S.C. 241(d); 5 U.S.C. 552a; 44 U.S.C. 3501 et seq.

## Supporting information

S1 Table Clonal Complex of Meningococcal Disease Isolates by Serogroup and Year, United States, 2020–2023. (DOCX)

S2 Table Outbreaks of meningococcal disease —United States, 2020–2023. (DOCX)
