# Supplementary material for: Meningococcal disease epidemiology – United States, 2020–2023
Source: PLoS One. 2026 Aug 31;21(8):e0356184. doi: 10.1371/journal.pone.0356184 (PMC13528998; doi:10.1371/journal.pone.0356184)
Supplement: Supplementary file 1 [file pone.0356184.s001.docx]

**Supplemental Table 1**. Clonal Complex of Meningococcal Disease Isolates by Serogroup and Year, United States, 2020–2023

|  | 2020  (n=153) | 2021  (n=127) | 2022  (n=202) | 2023  (n=345) |
| --- | --- | --- | --- | --- |
| CC* | N (%^†^) | N (%^†^) | N (%^†^) | N (%^†^) |
| Serogroup B |  |  |  |  |
| CC41/44 | 22 (57.9) | 11 (39.3) | 13 (31.0) | 14 (36.8) |
| CC32 | 4 (10.5) | 9 (32.1) | 12 (28.6) | 8 (21.1) |
| CC11 | 0 (0) | 0 (0) | 3 (7.1) | 6 (15.8) |
| Other | 12 (31.6) | 8 (28.6) | 14 (33.3) | 10 (26.3) |
| Serogroup C |  |  |  |  |
| CC103 | 28 (65.1) | 28 (49.1) | 25 (32.9) | 19 (47.5) |
| CC11 | 11 (25.6) | 18 (31.6) | 44 (57.9) | 14 (35) |
| CC35 | 1 (2.3) | 6 (10.5) | 0 (0) | 0 (0) |
| Other | 3 (7) | 5 (8.8) | 7 (9.2) | 7 (17.5) |
| Nongroupable (NG) |  |  |  |  |
| CC41/44 | 4 (22.2) | 3 (23.1) | 10 (41.7) | 8 (20.5) |
| CC32 | 4 (22.2) | 3 (23.1) | 2 (8.3) | 11 (28.2) |
| CC23 | 2 (11.1) | 2 (15.4) | 2 (8.3) | 7 (18) |
| Other | 8 (44.4) | 5 (38.5) | 10 (41.7) | 13 (33.3) |
| Serogroup W |  |  |  |  |
| CC11 | 10 (83.3) | 2 (50) | 9 (100) | 15 (100) |
| CC22 | 2 (16.7) | 2 (50) | 0 (0) | 0 (0) |
| Other | 0 (0) | 0 (0) | 0 (0) | 0 (0) |
| Serogroup Y |  |  |  |  |
| CC174 | 3 (7.3) | 1 (4) | 33 (64.7) | 148 (69.8) |
| CC23 | 32 (78.1) | 21 (84) | 16 (31.4) | 62 (29.3) |
| CC167 | 4 (9.8) | 2 (8) | 1 (2) | 2 (0.9) |
| Other | 2 (4.9) | 1 (4) | 1 (2) | 0 (0) |
