# Supplementary material for: Meningococcal disease epidemiology – United States, 2020–2023
Source: PLoS One. 2026 Aug 31;21(8):e0356184. doi: 10.1371/journal.pone.0356184 (PMC13528998; doi:10.1371/journal.pone.0356184)
Supplement: Supplementary file 2 [file pone.0356184.s002.docx]

**Supplemental Table 2**. Outbreaks of meningococcal disease —United States, 2020–2023.

| Outbreak Type | Year | Serogroup | Cases (deaths) | Duration (days) | Median age in years (range) | Clonal complex/sequence type |
| --- | --- | --- | --- | --- | --- | --- |
| Community-PEH | 2020 | B | 2 (0) | 2 | 40 (23-57) | CC41/44 / ST5981 |
| Organization-Other | 2021 | W | 2 (1) | 52 | 69.5 (64-75) | CC22 / ST2254 |
| Community-PEH | 2021 | C | 3 (3) | 6 | 32 (22-37) | CC11 / ST11 |
| Community-MSM | 2021–2023 | C | 45 (9) | 488 | 32 (9-77) | CC11 / ST11 |
| Community-PEH | 2022 | C | 3 (0) | 4 | 41 (37-71) | CC103 / ST2006 |
| Organization-University | 2022 | B | 3 (0) | 38 | 19 (4-20) | CC11 / ST11 |
| Community-Statewide* | 2022–2025 | Y | 41 (8) | 837 | 47 (16-80) | CC174 / ST1466 |
| Community-Other | 2022–2023 | W | 14 (3) | 370 | 43 (0-75) | CC11 / ST11 |
| Community-Other^†^ | 2023–2024 | B | 6 (0) | 37 | 2 (1-10) | CC41/44 / ST5981 |

*This outbreak in Virginia was declared over in February 2025. 32 cases and 6 deaths were reported during 2020–2023. Cases, deaths, duration and median age are calculated based off the entire outbreak.

^†^5 cases were reported during 2020–2023, and 1 additional case was reported in January 2024. Cases, deaths, duration and median age are calculated based off the entire outbreak.
